# The Solutions in Health Analytics for Rural Equity Across the Northwest (SHARE-NW) Dashboard for Health Equity in Rural Public Health: Usability Evaluation

**DOI:** 10.2196/51666

**Published:** 2024-06-05

**Authors:** Elizabeth Heitkemper, Scott Hulse, Betty Bekemeier, Melinda Schultz, Greg Whitman, Anne M Turner

**Affiliations:** 1 School of Nursing The University of Texas at Austin Austin, TX United States; 2 School of Medicine Uniformed Services University of the Health Sciences Bethesda, MD United States; 3 School of Nursing University of Washington Seattle, WA United States; 4 School of Public Health University of Washington Seattle, WA United States; 5 School of Medicine University of Washington Seattle, WA United States

**Keywords:** data dashboard, rural health, health equity, usability, nursing informatics, dashboard, rural, informatics, satisfaction, think aloud, content analysis, user experience, public health, visualization, information systems

## Abstract

**Background:**

Given the dearth of resources to support rural public health practice, the solutions in health analytics for rural equity across the northwest dashboard (SHAREdash) was created to support rural county public health departments in northwestern United States with accessible and relevant data to identify and address health disparities in their jurisdictions. To ensure the development of useful dashboards, assessment of usability should occur at multiple stages throughout the system development life cycle. SHAREdash was refined via user-centered design methods, and upon completion, it is critical to evaluate the usability of SHAREdash.

**Objective:**

This study aims to evaluate the usability of SHAREdash based on the system development lifecycle stage 3 evaluation goals of efficiency, satisfaction, and validity.

**Methods:**

Public health professionals from rural health departments from Washington, Idaho, Oregon, and Alaska were enrolled in the usability study from January to April 2022. The web-based evaluation consisted of 2 think-aloud tasks and a semistructured qualitative interview. Think-aloud tasks assessed efficiency and effectiveness, and the interview investigated satisfaction and overall usability. Verbatim transcripts from the tasks and interviews were analyzed using directed content analysis.

**Results:**

Of the 9 participants, all were female and most worked at a local health department (7/9, 78%). A mean of 10.1 (SD 1.4) clicks for task 1 (could be completed in 7 clicks) and 11.4 (SD 2.0) clicks for task 2 (could be completed in 9 clicks) were recorded. For both tasks, most participants required no prompting—89% (n=8) participants for task 1 and 67% (n=6) participants for task 2, respectively. For effectiveness, all participants were able to complete each task accurately and comprehensively. Overall, the participants were highly satisfied with the dashboard with everyone remarking on the utility of using it to support their work, particularly to compare their jurisdiction to others. Finally, half of the participants stated that the ability to share the graphs from the dashboard would be “extremely useful” for their work. The only aspect of the dashboard cited as problematic is the amount of missing data that was present, which was a constraint of the data available about rural jurisdictions.

**Conclusions:**

Think-aloud tasks showed that the SHAREdash allows users to complete tasks efficiently. Overall, participants reported being very satisfied with the dashboard and provided multiple ways they planned to use it to support their work. The main usability issue identified was the lack of available data indicating the importance of addressing the ongoing issues of missing and fragmented public health data, particularly for rural communities.

## Introduction

Data visualization dashboards developed to address health and equity have become increasingly popular [1,2]. Leveraging the longstanding history of using dashboards to aggregate and analyze data in public health [3] and medicine [4], these new dashboards cover myriad health equity–focused topics and target broad audiences. Recently, Thorpe and Gourevitch [5] identified 15 examples of US-based health dashboards that illustrate this growing trend. Examples range from a COVID-19 dashboard that highlights inequities in cases and deaths by geography to a policy dashboard that aggregates local laws and policies that affect population health [5]. Similar to these dashboards, the solutions in health analytics for rural equity across the northwest (SHARE-NW) dashboard (SHAREdash) was created to address health equity for rural communities.

Delivery and allocation of health services through public health agencies is a key mechanism for achieving health equity in the United States as they provide health prevention and promotion services and care [6]. Nationally, people in rural and frontier jurisdictions have significant health disparities compared with urban populations but are frequently the least well served by their public health agencies—local health departments (LHDs) [7,8]. Exacerbating this is the poor public health data systems, as updating to include information on structural and social factors has not been a top priority in LHDs’ activities or spending [8,9]. Research has highlighted the critical need to improve timely and reliable population health data to inform resource allocation and decision-making [10-14]. Consequently, decisions regarding the delivery of public health services and care primarily rely on conventional wisdom. This results in services that frequently do not reflect the needs of the populations they serve resulting in wasteful, harmful, and inequitable inefficiencies that exacerbate existing disparities [15-17]. To address these issues and support LHDs serving rural areas, the goal of SHAREdash is to provide accessible and relevant data that will enable public health professionals to identify, communicate, and address health disparities in their jurisdictions and with their communities. Developed with user-centered participatory design methods and guided by Munzer’s Nested Model for Visualization Design and Validation [18], SHAREdash is the first rigorously designed health equity dashboard developed for rural communities that we are aware of [19].

While clear objectives and thoughtful design are critical to ensuring the development of useful dashboards, Thorpe and Gourevitch [5] highlight the importance of evaluating dashboards and the need for a more rigorous assessment of the effectiveness and usefulness of health equity dashboards. Evaluating the performance of a dashboard through end user usability testing is a critical and often missed component of dashboard creation. The International Organization for Standardization defines usability as “the extent to which a product can be used by specified users to achieve specified goals with effectiveness, efficiency, and satisfaction in a specific context of use” [20,21]. Poor usability has been shown to increase errors [22-24], increase the time to complete tasks [25], and reduce user uptake [26-28] and implementation efforts [29].

Proper assessment of technology usability should occur at multiple stages throughout the system development lifecycle (SDLC) and use the methods most appropriate for that respective stage. In a review of usability study methodologies of health information technology by Yen and Bakken [30], the authors outline the importance of conducting multiple usability evaluations that align with the 5 stages of the SDLC. Furthermore, the Yen and Bakken [30] review clarifies the differences in usability evaluation types and goals based on the SDLC stage of the technology (Multimedia Appendix 1). Results from SHAREdash’s usability testing for SDLC stages 1 and 2 have been previously published [10,19]. Stage 2 findings were used to make critical changes and inform dashboard completion. Now that SHAREdash is finished and has entered SDLC stage 3, we evaluated its usability by examining all components combined (ie, the finished dashboard). Thus, the aim of this study was to evaluate the SDLC stage 3 evaluation goals of efficiency, satisfaction, and validity for SHAREdash.

## Methods

### The SHARE-NW Project and Dashboard

SHARE-NW is a partnership research project that was created with the goal of making data available and accessible to rural LHD practitioners, while building their capacity for data use and data-driven decision-making [10]. Partnering with LHDs in Alaska, Idaho, Oregon, and Washington, 7 priority topic areas (obesity, diabetes, tobacco use, mental and behavioral health, violence and injury prevention, oral health, and demographics) were identified during stage 1 of the SDLC for SHAREdash [19]. Data for the dashboard come from 36 unique data sources, including national data from the Centers for Disease Control and Prevention as well as local agencies and health departments (Multimedia Appendix 2). Data were deemed relevant to be included in the dashboard if it (1) addressed 1 of the 7 priority areas and (2) was provided at the county level so that it would be relevant to LHDs. To ensure the dashboard is usable and relevant for users, its features (eg, dynamic filters, pop-up tooltips, and visualizations) were created in collaboration with the staff from partner LHDs during SDLC stage 2. SHARE-NW has also developed a curated repository of web-based trainings and webinars, including new training modules developed in 2021 and launched in 2022 when gaps were found in the related training desired by practitioners. The new training modules developed use problem-based learning to teach audiences how to use and communicate data to promote health equity.

After conducting a needs assessment with rural LHD professionals during SDLC stage 1 [10], members of the SHARE-NW team identified a set of initial design requirements for SHAREdash. These requirements guided design and development decisions that ranged from key decisions, such as the selection of the best software to create the dashboard, to smaller decisions such as which size font to use for a graph label. Together with the findings from the SDLC stage 2 usability study, SHAREdash was completed and launched in August 2021.

### SHAREdash Website and Interface

**Figure 1:**
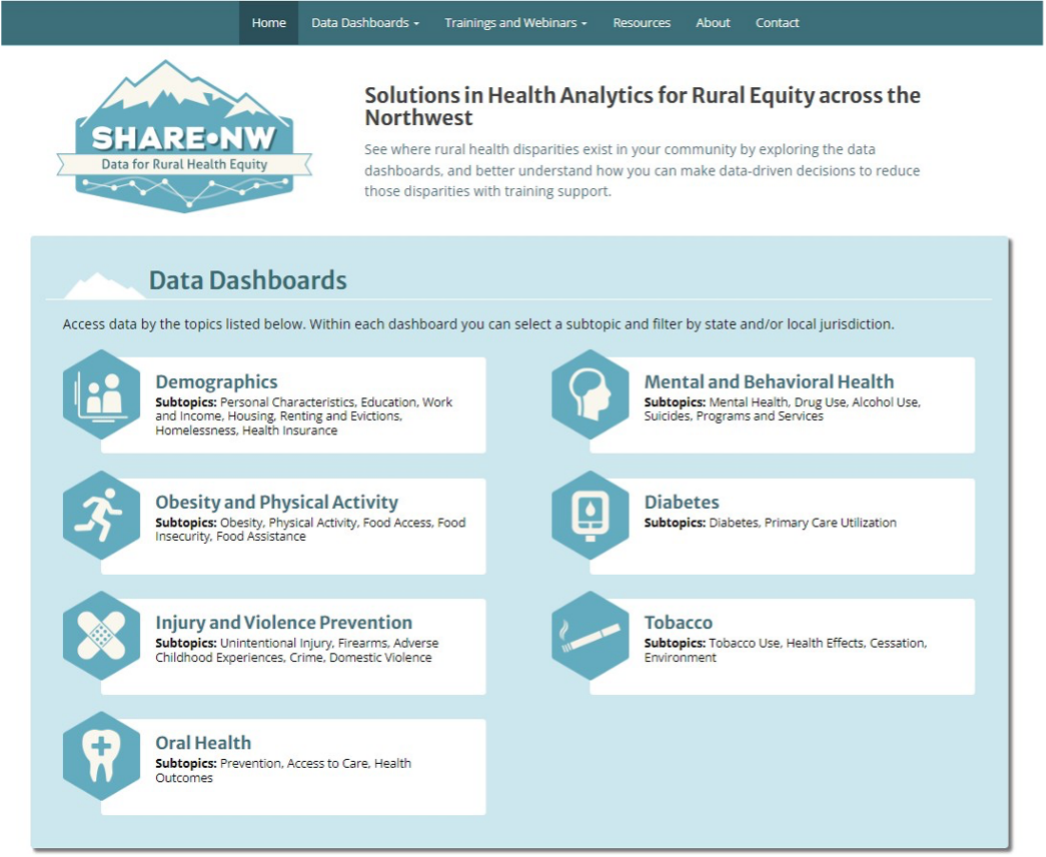
The solutions in health analytics for rural equity across the northwest dashboard (SHAREdash) home page.

SHAREdash is a Tableau-based dashboard with a header at the top of the main page for users to locate information about the project and team, access resources on relevant topics such as data, communication, and health equity, how to contact a member of the team, and find the dashboards organized by priority topic area. Users can also see relevant trainings and webinars (both via drop-down boxes) on SHAREdash’s main page. When users scroll down the main page, they can also find information on the website’s purpose and design and see the sources of data powering the website. The largest feature on the main page (Figure 1) links to the 7 dashboards on the priority topic areas mentioned previously [19]. Within each dashboard, users can find state and county-level data organized by relevant subtopics. For example, the topic of “Tobacco” includes subtopics of “Tobacco use,” “Health effects,” “Cessation,” and “Environment.”

When users navigate to each of the main topics, they find a header that lists the main topic and each subtopic along the top, such that users can click through them. Within each subtopic, there are several drop-downs that allow users to filter the data. The primary drop-down prompts the user to “Select an Indicator.” Some examples of indicators for the topic of “Tobacco” and the subtopic of “Tobacco use” are as follows: “8^th^ graders’ current tobacco use include Percent %,” “high school students who smoked tobacco in the past month: Percent %,” “Adults who currently smoke, Age-adjusted Percent %,” and “Current e-cigarette use: Percent %.” The remaining drop-downs allow users to filter the results by state, region, rurality (eg, rural or not rural), and jurisdiction types (eg, county). Users can export any of the dashboard views using the 3 options of export image, export to PDF, and share a link that is found along the top of the page. Along the bottom of each dashboard is the clickable link to view the data sources for this dashboard along with a statement explaining which data are listed as unavailable data within the dashboards.

### Study Setting and Participants

To evaluate this web-based dashboard, our study was conducted from January to April 2022. Participants were recruited from the states upon which SHAREdash was focused—Washington, Idaho, Alaska, and Oregon. All individuals who were rural public health professionals or trainees and had completed at least 1 prior SHARE-NW activity (2017-2022) and agreed to be contacted for future research activities (n=20). Prior SHARE-NW activities included the following: key informant interviews, interviews about the response to COVID-19, web-based surveys, dashboard mock-up testing sessions, dashboard usability testing activities, and group-based dashboard live training sessions. This ensured that all participants met the eligibility criteria of being at least 18 years old, working in public health for at least a year, and were in 1 of the 4 northwest states included in SHAREdash. Given public health differences by state, recruitment efforts ensured that at least 3 of the states were represented. Recruitment of this convenience sample had no additional inclusion or exclusion criteria.

### Ethical Considerations

The University of Washington’s institutional review board (IRB) approved the study protocol before participant recruitment (STUDY00013451). This study’s IRB was approved as a modification to an original approval (MOD00011747; approved on December 13, 2021).

An initial recruitment email briefly summarizing the study purpose was sent to all prior participants in SHARE-NW activities. A follow-up recruitment email was sent 2 weeks after the initial email. The response rate for recruitment was 45% (n=9) with 1 person stating that they did not want to participate and the remaining 10 people not responding. Individuals who expressed interest were sent an email with information about the study, consent to participate, and instructions on scheduling their interview. Dashboard evaluation sessions consisted of 2 think-aloud tasks [31] and open-ended interview questions [32] regarding the participant’s occupation and perceptions of the dashboard (Multimedia Appendices 3 and 4). Recruitment stopped when the following metrics were reached: (1) alignment with the published literature on the minimum number of participants needed to identify usability issues [33] and (2) when data saturation was reached. Given how stretched our public health partners were from the COVID-19 pandemic, the study team was cautious to not overburden them with study participation requests.

The think-aloud tasks served 2 purposes—the first was to refamiliarize the participant with SHAREdash and the second was to examine the usability components of effectiveness and efficiency. The first think-aloud task had the participant complete a simple task that consisted of switching between different subtopics, filtering for the participant’s county, and changing the time frame being viewed. The second, more complex task included navigating to the right topic, filtering for a specific health outcome type, year, and rate, and identifying the original sources of the data being viewed. During testing, the moderator prompted participants to “think aloud” that is, verbalize their thoughts as they worked through the task. Following the tasks, the participants completed qualitative interviews that asked participants for their perceptions regarding SHAREdash’s efficiency, validity, and satisfaction. The semistructured interview guide included questions that asked about the design aesthetics and functionality of SHAREdash, how quickly they are able to perform tasks, and the benefits and issues with using SHAREdash. The evaluation sessions were completed and recorded via a videoconferencing platform since screen sharing was needed for the 2 think-aloud task evaluations. Transcripts were automatically generated by the videoconferencing platform and stored securely in a password-protected cloud-based repository. A member of the research team deidentified and corrected any errors in the verbatim transcripts prior to analysis.

### Recruitment and Data Collection

#### Think-Aloud Task Analysis

Operational definitions of the outcomes align with the International Organization for Standardization definitions of efficiency, satisfaction, and validity [34]. Efficiency and validity were primarily evaluated through the think-aloud tasks. The number of clicks taken to complete the task indicated efficiency and data on the participants’ success of task completion were operationalized as “yes” (eg, no assistance needed), “no” (eg, assistance needed), or “partial” (eg, where the moderator confirms participant choices as either correct or incorrect but offers no other assistance) which indicated validity. Task 1 could be completed in a minimum of 7 clicks and task 2 could be done in 9 clicks. Transcripts were automatically generated and edited by a member of the research team for accuracy. Descriptive statistics were used to analyze the data and was completed in Excel. Quotes from the think-aloud tasks were analyzed to evaluate common efficiency issues, examine overall satisfaction, and assess validity. A control arm was not used in this study based on prior work that identified the inability of participants to complete these tasks without SHAREdash [10,19].

#### Qualitative Analysis

Data analysis of qualitative interview transcripts started with a directed, deductive approach to content analysis that was guided by a codebook comprising the initial codes of efficiency, satisfaction, and validity [35]. From this initial schema, iterative coding categories emerged as themes were developed. Coding was performed in NVivo (Lumivero) to organize data and provide an audit trail. Our interdisciplinary team of researchers met for an initial 90-minute collaborative coding session to talk through coding procedures and develop consensus for initial categories. Subsequent coding was performed independently with researchers meeting for 60-minute coding meetings to discuss categories and resolve discrepancies. Procedures for ensuring credibility, transferability, dependability, and confirmability were incorporated throughout the research process to ensure data trustworthiness. These procedures included taking field notes, team debriefing, reflexive journaling, consideration of negative cases, and maintenance of an audit trail. Data saturation was reached with researchers initially identifying the potential for saturation after the sixth participant interview and later confirming it with the ninth and final participant.

## Results

### Overview

Interviews lasting an average of 21 (SD 5.4) minutes were conducted between January and April 2022. Of the 9 public health practitioners interviewed, 4 were from Idaho, 3 were from Oregon, and 2 were from Washington (Table 1). Participants all identified as female, and the majority worked for health departments (n=8). Job positions included a director (n=2), managers (n=2), program specialists/coordinators (n=3), an epidemiologist (n=1), and a student/public health intern (n=1). Prior experience with Tableau was minimal with the majority (7/9, 78%) reporting less than 3 months experience to no experience.

**Table 1:** Demographics of dashboard evaluation participants (N=9).

Characteristic		Values, n (%)
**State**		
	Idaho	4 (44)
	Oregon	3 (33)
	Washington	2 (22)
**Organization type**		
	Local health department	7 (78)
	State health department	1 (11)
	Educational institution	1 (11)
**Position**		
	Director	2 (22)
	Coordinator	2 (22)
	Manager	2 (22)
	Other	3 (33)
**Tableau experience**		
	0-3 months	7 (78)
	>3 months	1 (11)
	Not reported	1 (11)
**Sex**		
	Female	9 (100)
**Ethnicity**		
	Not reported	5 (56)
	White	4 (44)

### Think-Aloud Task Results

For efficiency, mean clicks were 10.1 (SD 1.4) for task 1 (with a minimum of 7 clicks) and 11.4 (SD 2.0) for task 2 (with a minimum of 9 clicks; Table 2). For task 1, extra clicks occurred when people tried to find the right place to filter for the correct dashboard page. One participant (participant 9) required partial assistance with one of the steps in the first task. They initially thought to navigate to the default subtopic of “Personal Characteristics” instead of the correct subtopic “Homelessness” in the “Demographics” dashboard. Although only 1 participant required assistance with this step, many participants took extra time with it. For task 2, extra clicks resulted from people looking for the sources of the data, which were located at the footer of each dashboard. Most participants were able to find the “View Data Sources” button easily because of the dashboard’s instructions or because it was where they expected it based on their prior experience. However, 3 participants noted that they naturally scrolled to the bottom of the dashboard looking for it but confused the “Resources” button with the “View Data Sources” button. This issue not only resulted in extra clicks but also was the point where these participants required confirmation by the moderator to continue. Of the 3 participants, 2 who had this issue stated that they were confused because they expected the data sources to be in a clickable pop-up or an in-text citation, rather than loading onto a separate page.

**Table 2:** Efficiency task analysis results (N=9).

Task type			Task 1	Task 2
**Efficiency**				
	Clicks, mean (SD)	10.1 (1.4)		11.4 (2.0)
	Clicks, median (IQR)	10.5 (9.0-11.0)		11.0 (9.5-12.8)
**Validity, n (%)**				
	Successful	8 (89)		6 (67)
	Partial	1 (11)		3 (33)
	Not successful	0 (0)		0 (0)

Validity scores for both tasks were high with all participants (n=9) able to complete each task accurately and comprehensively. Most participants received a “yes” indicating that they did not require any prompting—89% (n=8) participants for task 1 and 67% (n=6) participants for task 2. For participants who did not receive a score of “yes,” they only required the moderator to either confirm or deny their decisions prior to moving on, resulting in a score of “partial.” None of the participants received a “no,” indicating they could not finish the tasks.

### Qualitative Results

The following 4 themes regarding efficiency were identified: “using the best terms and names to increase efficiency,” “drop-down filters reduce efficiency,” “minor navigation issues affect efficiency,” and “learnability will increase efficiency over time”. The primary issue that came up in the think-aloud tasks and the interviews was related to the dashboard labels and names that informed the theme of “using the best terms and names to increase efficiency.” Multiple participants brought up the term “jurisdiction” and pointed out that it is less intuitive than the word “county.” “I think ‘jurisdictions’ is obviously not wrong; it just would be a little bit more user-friendly to label it ‘county’” (participant 1). Similarly, as identified in the second task analysis, 2 participants found the “Resources” button confusing and suggested renaming it to something more specific to mitigate this confusion.

For the second theme of “drop-down filters reduce efficiency,” participants described how the functionality of the filters was difficult to navigate between some options due to the length of the drop-down boxes. For example, filtering to examine a single county requires users to search down a long drop-down list for the exact county they are looking for. Participants described this by saying, “Maybe it would be a nice feature to be able to type in a county versus the drop-down box or having to—well I guess you can ‘select all’ so you don’t have to go through and click them all to select, but just those little things might make it easier” [participant 4]. Another participant described how they expected the interface to be like other software they are used to using such that it allows users to enter free text into a search bar and then, “…when you start typing things it only picks the things that match it” (participant 8).

For the third theme of “minor navigation issues affect efficiency,” a few participants had difficulty locating the various subtopics within a dashboard, despite them being listed underneath each dashboard topic. For example, 1 participant looked for the “homelessness” indicator under the wrong subtopic.

I was initially thinking, ‘Oh ‘Homelessness’ must be in one of these drop downs, because it was listed as a subtopic,’ but then I glanced across the screen, and—you know—I saw ‘Homelessness’ up in this corner [with the other subtopics].

participant 2

Similarly, 3 participants eventually correctly identified that “Demographics” was the dashboard where they would find homelessness data, but they initially looked for the “homelessness” indicator under the “Housing” subtopic instead of the “Homelessness” subtopic. A participant suggested ways that the design of SHAREdash could be updated to more clearly indicate the subtopics.

I would think that [the indicator] is definitely going to be in Oral Health. It took a bit when I first looked at [SHAREdash] to realize that there were tabs (e.g., different subtopics). I think the size of the font and the fact that they are the same color as the bar makes it, so they are not standing out.

participant 1

These design suggestions were checked with some subsequent participants who agreed that changing the font size and color would help the subtopics stand out.

For the final theme of “learnability will increase efficiency over time,” participants spoke about how quickly they were able to figure things out in SHAREdash and reported that with repeated use they thought they would quickly improve over time. Half of the participants stated that first-use learnability was high such that SHAREdash was easy to use the first time they tried. “I would say that there’s not a lot of websites out there, where you can pick up on things that quickly. So, I immediately don’t have any areas for improvement” [participant 2]. Whereas the remaining half of the participants stated that they felt like they would get progressively better at using SHAREdash over time.

The following 3 themes related to overall satisfaction were identified: “high potential to support work,” “enables meaningful comparisons,” and “needs more up-to-date data.” For the theme of “high potential to support work,” participants spoke positively about how much they liked SHAREdash and the myriad ways they could use SHAREdash’s various features to support their work. One-third of the participants mentioned how unique and helpful it was to have the ability to export and share graphs.

I think it has a lot of features that aren’t necessarily easily found [in other dashboards]…blowing it [SHAREdash] up to full screen, downloading it, sharing it—that’s not necessarily common with dashboards, so I appreciate that…It could be really useful for like a grant application or demographic reporting for part of a program.

participant 5

Two participants mentioned that they might direct others to the dashboard so they could interact with data, and this was described as something that would be “extremely useful” and “super helpful” in their work. Finally, several participants identified specific types of work that SHAREdash would meaningfully support such as completing community health assessments:

We would definitely want to look at this in relation to the approach that we took with our CHA [community health assessment]. Most recently, I was trying to mine all of the data sources that are already in existence to inform it and see where some gaps were, and then we did primary data seeking based off those gaps instead of trying to reproduce data that’s already in existence. And so, this [SHAREdash] would be a really great one-stop-shop to look at a lot of different ones at one time.

participant 2

I think it is already something that’s on our radar when we talk about this CHA [community health assessment] that I mentioned. So, we’re not here to duplicate efforts; let’s use what’s out there. And so, we’ll probably refer to it [SHAREdash] for that.

participant 5

For the theme of “enables meaningful comparisons,” all but 2 participants reported that they were highly satisfied with SHAREdash and cited the ability to compare their county or region with other neighboring or similar counties in different states as the reason why. Multiple participants stated they wanted to look at counties in nearby states given their close proximity and described how SHAREdash fills this gap since states do not typically share data with one another.

Being able to look at data kind of in the same place and say ‘Oh, what does your county look like?’ You know, which borders us in Oregon, but borders like three of the counties that I oversee. So, what’s happening in their county? I can look that up and see if we’re seeing similar trends, and the three counties that border that county. So having that originality, I think, is great and is probably a reason that I would go to the website to look at that at some point, or my team would.

participant 9

It is nice that it includes multiple states, because we are border county in our state, and so a lot of times things that we see are only for Oregon. But we’re right next to a couple of Washington counties and it would be great to, you know, compare in that manner as well…It’s always really helpful when we can look at, you know, what is our information compared to our neighboring counties, what does our data look like compared to counties of similar size.

participant 2

I like that you can see a big picture, regionally. So not necessarily just like other counties in Idaho: being able to prepare to other regional and other states and perhaps similar geographic demographic areas that are comparable, but in different states kind of just to see what trends are like there comparatively.

participant 4

The third theme of “needs more up-to-date data” described the biggest challenge that participants identified to their overall satisfaction with SHAREdash.

I think just what I commented on already is the age of the data that is present. So, it is very difficult to make a decision on data that’s extremely outdated. And it’s hard to make it relevant to your case. And I know that data can be hard to gather and hard to access, but for those of us who are looking at data to make decisions, that complicates that entire scenario. You want us to use data to make decisions, we need good data to make those decisions. Somebody has to put the data out.

participant 6

Several participants acknowledged that none or out-of-date data are typical within public health, particularly for rural areas. “We’re used to that, so I think for us that’s not a missed expectation to click on it and be like ‘Oh, there’s not any new data.’…That for us, that’s normal” [participant 2]. However, this is a clear barrier to satisfaction and future use of SHAREdash.

There were 2 themes identified on validity called “reputable data sources increases validity” and *“*impact of missing data decreases validity.” Participants spoke about the second theme of “reputable data sources increases validity” by describing their confidence in the data quality and accuracy. A participant described this by stating that, “SHAREdash is a really amazing place to quickly get domestic violence rates across other states. And you can find the source easily. And it is a reputable source too” [participant 1].

Whereas, another participant emphasized more than just the high quality of the data sources, but also the fact that SHAREdash’s team provided a second, external check on it:

So I think this is a great dashboard and it’s so nice because part of my job is to pull [data] from all of these different data sources which I know SHARE has done, and it’s been validated and checked and it’s a combination of information from various places which is good to have.

participant 3

For the “impact of missing data decreases validity” theme, task 1 had participants refined the population of interest to their specific county, which for some participants resulted in SHAREdash indicating that there were no data for their respective county available. Participants described how missing data in the dashboard impacted their ability to completely address the tasks in the think-aloud evaluation and how it would impact their work.

I think one of my biggest challenges, and it tends to be a challenge everywhere not just like solely for the dashboards, is that a lot of times when there were things I wanted to look at and there wasn’t any data available because our population isn’t that big.

participant 8

Despite multiple participants acknowledging that problems with data availability for rural areas is a known issue and is not a fault of the dashboard, they still expressed frustration and dissatisfaction about this issue.

I was bummed when it didn’t have the data that I was looking for. But like I said, it’s probably just a result of that data not being available.

participant 4

## Discussion

### Principal Findings

This SDLC stage 3 usability evaluation of SHAREdash, a dashboard designed for rural public health, indicates that overall SHAREdash is an efficient and valid tool that users reported being satisfied with. Task analyses and qualitative findings illustrate how SHAREdash’s collaborative co-design process resulted in a tool that is easy to use and supports rural public health professionals’ work. Thematic results also identified areas where SHAREdash can be improved to increase its usability such as changing some of the terms and names used and considering alternate ways for users to view and select information that are not just drop-down filters. However, this evaluation also uncovered usability issues related to the lack of public health data that go beyond design aspects and cannot be addressed through modifying SHAREdash’s interface or navigation.

Issues related to obtaining quality public health data are well documented in the literature and include the critical problems of a lack of investment in public health data systems and infrastructure [36-39], issues with data quality [40-42] and data fragmentation [43,44], and the sparse data available about rural communities [8,45]. While every effort was made to include as much timely and comprehensive data as possible in SHAREdash, these larger data problems clearly impacted the usability of this tool. Thus, returning to the question posed by Thorpe and Gourevitch [5] regarding whether or not data dashboards for advancing health and equity are fulfilling their promise, findings from our study show that, to fully realize the potential of health equity–focused dashboards substantial investments in public health data need to be made. Unlike health care which benefited from the 2009 Health Information Technology for Economic and Clinical Health Act that supported the adoption of meaningful use of electronic health records [46,47], other systems, such as public health and social services, were not included in these incentives resulting in the numerous data issues seen today. The need for investments in public health data and systems was further clarified and reinforced by the COVID-19 pandemic which magnified many of these ongoing challenges [38,45,48]. To address this urgent problem, supportive policies that fund public health data collection and systems should leverage the successes of the Health Information Technology for Economic and Clinical Health Act and learn from the opportunities to address and alleviate these issues.

Another key finding from this study is also related to data. All the participants emphasized the significance of the trustworthiness of the data in SHAREdash. These results align with prior literature that has articulated the dual importance of dashboards to use data from reputable sources and clearly display or link to original data sources [49]. In a 2020 study by Young and Kitchin [50] that examined user perspectives of 4 different city’s dashboards to create design guidelines, the authors stipulate how critical the veracity (eg, accuracy, source, and age) of the included data is. Our findings reinforce this work and indicate the utility of their design guidelines for creating data dashboards of municipal data. Future dashboards of municipal data should use the guidelines provided by Young and Kitchin [50] in the early design and development stages and work with target users to refine them for their specific project needs.

Satisfaction with SHAREdash was high with most participants describing the usefulness of the dashboard in supporting their work. Almost all the participants reported that they would like to make local-level comparisons that cross their respective states and articulated how difficult this currently is. Participants reported how comparisons between counties across different states can be more meaningful than within if they are able to filter for key factors such as population size or number of services available and how helpful it is that SHAREdash facilitates this easily. Enabling such comparisons points to the importance of aggregating large amounts of data across states, particularly for rural health departments that have unique needs and face different challenges than their urban counterparts [51]. It also indicates the importance of continuing to elucidate the unique needs of rural public health. Future research should focus on rural public health so that tailored design guidelines and specialized tools can be developed to support their work in addressing health disparities.

Our SDLC stage 3 usability assessment indicated that SHAREdash is meeting the goal of providing accurate, accessible, and relevant data via a user-centered dashboard to address health equity for rural communities. Next steps for SHAREdash will focus on identifying the elements key to its integration into LHDs using an implementation science approach that is outlined in stage 4 of the SDLC [30]. Planning for this phase is underway and is working closely with future end users to proactively identify and understand barriers to integration as this was a clear lesson learned from a similar study implementing an ICU dashboard [52]. Furthermore, investing in efforts to understand what is needed to support the uptake of health equity–focused dashboards in public health practice is critical to ensuring their impact [5] and aligns with previously identified public health research priorities [53-55] that highlights the importance of using implementation science to translate and assess innovations into public health practice to ensure reach. It is hoped that through the user-centered development and thoughtful translation of informatics, tools such as SHAREdash will address the existing health disparities and improve rural health equity.

### Limitations

While our methods were rigorous, this study has limitations. Despite reaching data saturation, the sample size is small, consisting of all female-identifying participants, and limited to the northwest United States. Of note, the public health workforce is 79% women [56], which made diversity by sex difficult to obtain. Future studies would benefit from a larger and broader sample. Additionally, the think-aloud task analysis did not have a control arm where participants completed the tasks without SHAREdash to provide a comparison. Finally, while aligned with the SLC stage 3 usability evaluation components, this study did not examine other aspects of usability that are outlined in the literature, and thus, might have missed certain usability aspects [57].

### Conclusions

Evaluating the usability of health equity dashboards is crucial to creating effective and valuable tools. Our findings indicate that SHAREdash, a public health dashboard created to support promoting health equity among rural communities, is an efficient, valid tool that overall users are satisfied with. Results strongly suggest that the utility of dashboards such as SHAREdash would be improved with the availability of more public health data and supportive policies to achieve robust collection of public health data would be beneficial. Future research should continue to focus on building tools that meet the unique needs of professionals working in rural public health to better support and equip them to alleviate rural health disparities.

## Appendix

Multimedia Appendix 1 Usability evaluation types and goals based on the system development lifecycle (SDLC) stage (adapted from Yen and Bakken [30], which is published under Creative Commons Attribution-NonCommercial-NoDerivs licence).

Multimedia Appendix 2 Information on SHAREdash data sources.

Multimedia Appendix 3 Information on task analyses.

Multimedia Appendix 4 Semistructured interview guide.

## Abbreviations

IRB: institutional review board
LHD: local health department
SDLC: system development lifecycle
SHAREdash: solutions in health analytics for rural equity across the northwest dashboard
SHARE-NW: solutions in health analytics for rural equity across the northwest
