# Supplementary material for: The Solutions in Health Analytics for Rural Equity Across the Northwest (SHARE-NW) Dashboard for Health Equity in Rural Public Health: Usability Evaluation
Source: JMIR Hum Factors. 2024 Jun 5;11:e51666. doi: 10.2196/51666 (PMC11187519; doi:10.2196/51666)
Supplement: Multimedia Appendix 1 [file humanfactors_v11i1e51666_app1.docx]

Table S1. Usability evaluation types and goals based on the SDLC stage.

| Stage | Criteria | Goal |
| --- | --- | --- |
| Stage 1:  Specify needs for setting and users | Needs assessments with design methods described | - Describe definition/specifications |
| Stage 2:  System component development | System validation evaluations, such as sensitivity and specificity analyses, ROC curve, and observer variation | - Validity: accuracy, sensitivity and specificity, and speed |
| Stage 3: Combine components | Human-computer interaction evaluations focusing on outcome quality, user perception, and user performance in the laboratory setting | - Efficiency: speed and learnability - Satisfaction: user perception - Validity: accuracy and completeness |
| Stage 4:  Integrate system into setting | Field testing; experimental or quasi-experimental designs with control groups in one setting | - System effectiveness: accuracy, completeness, utilization, workflow - Efficiency: process speed, workflow efficiency - Satisfaction: user perception |
| Stage 5: Routine use | Field testing; experimental or quasi-experimental designs with control groups in multiple sites; post-implementation evaluation only; self-control, such as evaluation before and after implementation | - System effectiveness: accuracy, completeness, utilization, workflow - Satisfaction: user perception - Work efficiency: process speed, workflow efficiency - Work effectiveness:   - Practice pattern   - Prescribing behavior   - Cost–benefit analysis   - Quality of care   - Guideline adherence   - Patient outcomes   - Medication errors   - Communication/collaboration   - Provider-patient relationship   - Utilization |
|  | | |
