# Supplementary material for: The Solutions in Health Analytics for Rural Equity Across the Northwest (SHARE-NW) Dashboard for Health Equity in Rural Public Health: Usability Evaluation
Source: JMIR Hum Factors. 2024 Jun 5;11:e51666. doi: 10.2196/51666 (PMC11187519; doi:10.2196/51666)
Supplement: Multimedia Appendix 2 [file humanfactors_v11i1e51666_app2.docx]

Table S2. Information on SHAREdash data sources

| # | Data focus | Data source | Description | URL |
| --- | --- | --- | --- | --- |
| 1 | NL^1^ | Bureau of Alcohol, Tobacco, Firearms and Explosives | - The Bureau of Alcohol, Tobacco, Firearms, and Explosives publishes a list of Federal Firearms License holders. - The license allows individuals to engage in business related to the manufacture of ammunition or firearms or the interstate or intrastate sale of firearms. | https://www.americanbar.org/groups/public_education/publications/teaching-legal-docs/federal-firearms-licensing--an-overview/ |
| 2 | NL | CDC's My Water's Fluoride | - My Water's Fluoride allows consumers to learn about the fluoride level in their drinking water. | https://nccd.cdc.gov/DOH_MWF/Default/Default.aspx |
| 3 | NL | CDC National Environmental Public Health Tracking Network | - The National Environmental Public Health Tracking Network (Tracking Network) brings together health data and environmental data from national, state, and city sources and provides supporting information to make the data easier to understand. - The Tracking Network has data and information on environments and hazards, health effects, and population health. | https://ephtracking.cdc.gov/ |
| 4 | NL | CDC Opioid Overdoes U.S. Opioid Prescribing Rate Maps | - The data in the maps show the geographic distribution in the US, at both state and county levels, of retail opioid prescriptions dispensed per 100 persons per year from 2006–2020. | https://www.cdc.gov/drugoverdose/rxrate-maps/index.html |
| 5 | NL | County Health Rankings and Roadmaps | - The County Health Rankings & Roadmaps program provides data, evidence, guidance, and examples to build awareness of the multiple factors that influence health and support leaders in growing community power to improve health equity. | https://www.countyhealthrankings.org/about-us |
| 6 | NL | Dartmouth Atlas Project | - The Dartmouth Atlas of Health Care offers access to decades worth of Medicare data and supplemental materials including crosswalks and geographic boundary files. | https://data.dartmouthatlas.org/ |
| 7 | NL | Easy Access to State and County Juvenile Court Case Counts | - The National Juvenile Court Data Archive is a project maintained by the National Center for Juvenile Justice that collects data from juvenile courts across the country. - These data are used to develop national estimates of the delinquency and status offense cases handled by U.S. courts with juvenile jurisdiction and are the basis of the annual Juvenile Court Statistics series. | https://www.ojjdp.gov/ojstatbb/ezaco/asp/method.asp |
| 8 | NL | Eviction Lab | - The Eviction Lab at Princeton University has published the first ever dataset of evictions in America, going back to 2000. | https://evictionlab.org/about/ |
| 9 | NL | Federal Bureau of Investigation, Census Bureau | - The FBI's Crime Data Explorer aims to provide transparency, create easier access, and expand awareness of criminal, and noncriminal, law enforcement data sharing; improve accountability for law enforcement; and provide a foundation to help shape public policy with the result of a safer nation. | https://cde.ucr.cjis.gov/LATEST/webapp/#/pages/home |
| 10 | NL | Feeding America: Mapping the Meal Gap | - Feeding America conducts Map the Meal Gap annually to improve our understanding of how food insecurity and food costs vary at the local level. - The study estimates overall and child food insecurity for every county and congressional district in the United States as well as local food insecurity estimates for several racial and ethnic groups. | https://www.feedingamerica.org/research/map-the-meal-gap/by-county |
| 11 | NL | Kids Count Data Center | - The Annie E. Casey Foundation funds a nationwide network of state-level KIDS COUNT grantees to track the well-being of children in the United States. | https://datacenter.aecf.org/about |
| 12 | NL | Mapping Police Violence | - America's most comprehensive database of police violence. | https://mappingpoliceviolence.org/about |
| 13 | NL | NACCHO Profile Survey | - The National Association of County and City Health Officials conducts the National Profile of Local Health Departments (LHDs) every three years as a census of LHDs. - This represents the largest, most reliable data source on LHDs and describes the funding, staffing, governance, and activities of LHDs across the US, developing a comprehensive and accurate description of LHD infrastructure and practice. | https://www.naccho.org/resources/lhd-research/national-profile-of-local-health-departments |
| 14 | NL | US Cancer Statistics Data Visualizations Tool | - The official federal cancer statistics, produced by the Centers for Disease Control and Prevention and the National Cancer Institute. | https://gis.cdc.gov/Cancer/USCS/#/StateCounty/ |
| 15 | NL | US Census Bureau American Community Survey | - The American Community Survey is a nationwide survey that collects and produces information on social, economic, housing, and demographic characteristics about our nation's population every year. | ACS Information Guide - https://www.census.gov/content/dam/Census/programs-surveys/acs/about/ACS_Information_Guide.pdf |
| 16 | NL | US Census County Business Patterns and Nonemployer Statistics Combined Report | - The County Business Patterns and Nonemployer Statistics Combined Report, merges the published 2016 County Business data and the published 2016 Nonemployer Statistics data by industry to produce a more complete view of the economy. | https://www.census.gov/data/tables/2016/econ/cbp/2016-combined-report.html |
| 17 | NL | US Department of Agriculture Economic Research Service, Food Environment Atlas | - The current version of the Food Environment Atlas has more than 280 variables, including new indicators on food banks and nutrition assistance program participation rates. | https://www.ers.usda.gov/data-products/food-environment-atlas/data-access-and-documentation-downloads/ |
| 18 | NL | US EPA's National Walkability Index | - The National Walkability Index provides walkability scores based on a simple formula that ranks selected indicators from the Smart Location Database that have been demonstrated to affect the propensity of walk trips. | https://www.epa.gov/smartgrowth/smart-location-mapping#:~:text=The%20National%20Walkability%20Index%20is,to%20rank%20the%20block%20groups |
| 19 | AK^2^ | Alaska Department of Health and Social Services, Division of Public Health Vital Statistics | - The Bureau of Vital Statistics is responsible for issuing certified copies of vital records, including birth, death, marriage and divorce certificates for events that occurred in Alaska. | https://health.alaska.gov/dph/VitalStats/Pages/faqs.aspx |
| 20 | AK | Muck Boy OutDoors | - List of public shooting ranges and gun clubs in Alaska. | https://www.muck-boy.com |
| 21 | AK | University of Alaska Anchorage Justice Center | - The Justice Center is an interdisciplinary unit that provides undergraduate and professional education; conducts research in the areas of crime, law, and justice; and provides services to government units, justice agencies, and community organizations throughout urban and rural Alaska to promote a safe, healthy, and just society. | https://www.uaa.alaska.edu/academics/college-of-health/departments/justice-center/ |
| 22 | ID^3^ | Idaho Housing and Finance Association | - Idaho Housing and Finance Association is leading a coordinated effort to address the issues of homelessness in Idaho. - Outlined in the report are the interventions being carried out by Idaho Housing and partnering community programs to provide housing and support to displaced individuals and families. | https://www.idahohousing.com/homelessness-services-programs/idaho-homelessness-community-report/ |
| 23 | ID | Idaho Smile Survey, Bureau of Community and Environmental Health, Idaho Department of Health and Welfare | - The Idaho Department of Health and Welfare’s Oral Health Program contracts with the state’s seven Public Health Districts as part of its role to monitor and collect data on the oral health status of Idaho’s children and citizens. | https://publicdocuments.dhw.idaho.gov/WebLink/DocView.aspx?id=16180&dbid=0&repo=PUBLIC-DOCUMENTS |
| 24 | ID | Idaho Stat Rifle and Pistol Association | - List of shooting clubs (and places to shoot) around the state of Idaho. | https://idahosrpa.org/locations |
| 25 | ID | Idaho State Police | - Published crime rate data in Idaho. | https://isp.idaho.gov/pgr/sac/library/ |
| 26 | ID | Idaho Vital Statistic Reports | - The Idaho Vital Records system is a partnership between providers such as coroners, medical professionals, hospitals, funeral homes and the Bureau of Vital Records and Health Statistics to make sure of the completeness and accuracy of vital records for individuals and families. | https://healthandwelfare.idaho.gov/providers/vital-records/vital-records |
| 27 | OR^4^ | Oregon Department of Fish and Wildlife | - List of public shooting ranges and gun clubs published by Oregon Department of Fish and Wildlife. | https://www.oregonhuntingmap.com//#/disclaimer |
| 28 | OR | Oregon Department of Human Services | - Data about the Oregon Department of Human Services and data from the agency's central services and offices | https://www.oregon.gov/odhs/data/Pages/agency-data.aspx |
| 29 | OR | Oregon Health Authority | - The Oregon Health Authority Public Health Division collects and analyzes data on health behaviors, diseases and injuries, disseminates findings, and designs and promotes evidence-based programs and policies to improve the health and safety of all Oregonians. | https://www.oregon.gov/oha/PH/DATASTATISTICS/Pages/index.aspx |
| 30 | OR | Oregon Health Authority Deaths and Perinatal Deaths Data Annual Reports | - This report includes statewide mortality data from 2010-2021, offering visualizations for trends in leading causes of death. | https://www.oregon.gov/oha/PH/BIRTHDEATHCERTIFICATES/VITALSTATISTICS/ANNUALREPORTS/Pages/index.aspx |
| 31 | WA^5^ | State of Washington Department of Commerce | - Each year the U.S. Department of Housing and Urban Development and Washington state require a statewide count of all persons staying in temporary housing programs and places not meant for human habitation. | https://www.commerce.wa.gov/serving-communities/homelessness/annual-point-time-count/ |
| 32 | WA | University of Washington Alcohol and Drug Abuse Institute | - The Addictions, Drug and Alcohol Institute at the University of Washington is a focal point for alcohol and drug use research, benefiting the citizens of Washington State by expanding our knowledge and providing information to health and social service professionals, policy makers, and the public. | https://adai.uw.edu/about-us/ |
| 33 | WA | Washington Arms Collectors | - List of public shooting ranges and gun clubs published by Washington Arms Collectors. | https://washingtonarmscollectors.org/gun-range-finder/ |
| 34 | WA | Washington State Coalition Against Domestic Violence | - Domestic Violence Fatality Review provides up-to-date statistics for Washington State and research findings and recommendations for improving the community response to domestic violence. | https://wscadv.org/projects/statistics-research/ |
| 35 | WA | Washington State Department of Health | - Providing the latest data available on tobacco and vapor product use rates in Washington, including its health and economic burden. | https://doh.wa.gov/data-statistical-reports/health-behaviors/tobacco |
| 36 | WA | Washington State Department of Health Death Tables by Topic | - The Department collects high-quality information on all births, deaths, abortions, marriages, divorces, health factors, and hospitalizations that occur in Washington State. - We publish these data in tables to help data users understand trends, identify high risk populations and geographical areas, set prevention priorities, and plan health promotion strategies. | https://doh.wa.gov/data-and-statistical-reports/health-statistics |

^1^NL=National, ^2^AK=Alaska, ^3^ID=Idaho, ^4^OR=Oregon, ^5^WA= Washington
