# Supplementary material for: The Solutions in Health Analytics for Rural Equity Across the Northwest (SHARE-NW) Dashboard for Health Equity in Rural Public Health: Usability Evaluation
Source: JMIR Hum Factors. 2024 Jun 5;11:e51666. doi: 10.2196/51666 (PMC11187519; doi:10.2196/51666)
Supplement: Multimedia Appendix 3 [file humanfactors_v11i1e51666_app3.docx]

Table S3. Information on task analyses

| Assessment method | Evaluation component(s) | Operationalization | Task description |
| --- | --- | --- | --- |
| Simple think-aloud task | - Effectiveness - Efficiency | - Success of task completion (yes, no, partial) - Number of clicks to complete task | - Go to the “Demographics” dashboard   - Select 'Homelessness' from the ribbon   - Refine to just your county   - Select Multi-year view   Can you show me how many homeless people there are in your county over the last several years? |
| Complex think-aloud task | - Effectiveness - Efficiency | - Success of task completion (yes, no, partial) - Number of clicks to complete task | - Go to “Oral Health” dashboard   - - Select 'Health Outcomes' from the ribbon     - Select 'Adults 18+ with at least 1 permanent tooth removed: Age-adjusted percent (%)'     - Select multi-year view   How would you figure out why there might not be any data being displayed? |
