# Supplementary material for: The Solutions in Health Analytics for Rural Equity Across the Northwest (SHARE-NW) Dashboard for Health Equity in Rural Public Health: Usability Evaluation
Source: JMIR Hum Factors. 2024 Jun 5;11:e51666. doi: 10.2196/51666 (PMC11187519; doi:10.2196/51666)
Supplement: Multimedia Appendix 4 [file humanfactors_v11i1e51666_app4.docx]

Table S4. Semi-structured interview guide

| **Evaluation component** | **Interview question(s)** |
| --- | --- |
| Background | - Can you tell me about a time you used the dashboard since your initial training? If you haven’t used the dashboard since the training, can you talk about why? - If you used the dashboard since the training, was this related to your current job role and what was your workflow like? - How often are you using the dashboard or information you have obtained from the dashboard? - Have you ever shared any of the information from the dashboard with others? - If so, how did that go for you and if not, please elaborate on why? |
| Effectiveness | - How accurately do you think you were able to complete the tasks?   - Can you think of anything that could be changed to improve your ability to accurately complete the tasks? |
|  | - How completely do you think you were able to complete the tasks?   - Can you think of anything that could be changed to improve your ability to complete the tasks more fully? |
| Efficiency | - Please describe any problems or issues you encountered that made it hard to use the dashboard during the tasks. |
| Satisfaction | - How pleasant was it to use the dashboard? - Would you change anything about the dashboard design? - Please describe any benefits you’ve encountered using the dashboard.   - What do you like best about the dashboard? - Are there any features you would like to see added in the future? - Please describe any problems or issues you’ve encountered using the dashboard.   - What do you like least about the dashboard? - How can we improve the dashboard? |
